# Potassium Uptake Modulates *Staphylococcus aureus* Metabolism

**DOI:** 10.1128/mSphere.00125-16

**Published:** 2016-06-15

**Authors:** Casey M. Gries, Marat R. Sadykov, Logan L. Bulock, Sujata S. Chaudhari, Vinai C. Thomas, Jeffrey L. Bose, Kenneth W. Bayles

**Affiliations:** Department of Pathology and Microbiology, University of Nebraska Medical Center, Omaha, Nebraska, USA; University of Iowa

**Keywords:** *Staphylococcus aureus*, metabolism, potassium transport

## Abstract

As a leading cause of community-associated and nosocomial infections, *Staphylococcus aureus* requires sophisticated mechanisms that function to maintain cellular homeostasis in response to its exposure to changing environmental conditions. The adaptation to stress and maintenance of homeostasis depend largely on membrane activity, including supporting electrochemical gradients and synthesis of ATP. This is largely achieved through potassium (K^+^) transport, which plays an essential role in maintaining chemiosmotic homeostasis, affects antimicrobial resistance, and contributes to fitness *in vivo*. Here, we report that *S. aureus* Ktr-mediated K^+^ uptake is necessary for maintaining cytoplasmic pH and the establishment of a proton motive force. Metabolite analyses revealed that K^+^ deficiency affects both metabolic and energy states of *S. aureus* by impairing oxidative phosphorylation and directing carbon flux toward substrate-level phosphorylation. Taken together, these results underline the importance of K^+^ uptake in maintaining essential components of *S. aureus* metabolism.

**IMPORTANCE** Previous studies describing mechanisms for K^+^ uptake in *S. aureus* revealed that the Ktr-mediated K^+^ transport system was required for normal growth under alkaline conditions but not under neutral or acidic conditions. This work focuses on the effect of K^+^ uptake on *S. aureus* metabolism, including intracellular pH and carbon flux, and is the first to utilize a pH-dependent green fluorescent protein (GFP) to measure *S. aureus* cytoplasmic pH. These studies highlight the role of K^+^ uptake in supporting proton efflux under alkaline conditions and uncover a critical role for K^+^ uptake in establishing efficient carbon utilization.

## Abstract (precis)

Previous studies describing mechanisms for K^+^ uptake in *S. aureus* revealed that the Ktr-mediated K^+^ transport system was required for normal growth under alkaline conditions but not under neutral or acidic conditions. This work focuses on the effect of K^+^ uptake on *S. aureus* metabolism, including intracellular pH and carbon flux, and is the first to utilize a pH-dependent green fluorescent protein (GFP) to measure *S. aureus* cytoplasmic pH. These studies highlight the role of K^+^ uptake in supporting proton efflux under alkaline conditions and uncover a critical role for K^+^ uptake in establishing efficient carbon utilization.

## Observation

Adaptation to environmental change both within and outside the host is essential to *Staphylococcus aureus* pathogenesis. This can include adjustments to limited nutrient availability and a range of other environmental stresses. A number of recent reports describing mechanisms of potassium (K^+^) uptake and a role for K^+^ transport in *S. aureus* pathogenesis have shed light on the importance of K^+^ in *S. aureus* cell physiology and adaptation to stress (1–6). Various channels and dedicated transport systems are essential for bacteria to perform K^+^ uptake against steep transmembrane concentration gradients. General bacterial K^+^ requirements are often satisfied by moderate-affinity transporters, such as the Trk and Ktr systems, while high-affinity K^+^ transporters, such as the Kdp ATPase system, can be utilized under severe K^+^ limitation (7). The *S. aureus* genome encodes a unique Ktr-like system where the cytoplasmic gating protein KtrC (also termed KtrA) regulates the K^+^ uptake activity of the KtrB and KtrD channel proteins (1, 6). Interestingly, binding of the bacterial second messenger c-di-AMP to KtrC has been recently shown to inhibit Ktr-mediated K^+^ uptake (2, 4).

As the major intracellular cation, K^+^ plays a direct role in bacterial cell physiology and biochemistry, including the regulation of cell turgor pressure and governing the magnitude of the transmembrane electrical potential (ΔΨ) and cytoplasmic pH (8, 9). K^+^ uptake in exchange for proton (H^+^) extrusion is electroneutral and prevents ΔΨ hyperpolarization (outside positive); insufficient K^+^ uptake limits the number of H^+^ that can be released from the cell due to the ΔΨ that develops. As a result, the ΔΨ formed during H^+^ extrusion can be converted to a ΔpH (outside acidic) via K^+^ uptake, thus the ability of K^+^ to affect both components of the proton motive force (PMF). In acidic environments, the ΔpH of neutrophilic bacteria is relatively large (positive), and so the ΔΨ must be small, as H^+^ efflux would be inhibited by both electrical and chemical H^+^ gradients. To maintain a small or reversed ΔΨ, K^+^ influx must at least equal H^+^ efflux. As a result, bacteria typically rely on K^+^ transport for growth during acidic stress. In alkaline environments, however, the ΔpH is small or negative, and so H^+^ return for ATP synthase activity must be energized by a large ΔΨ. Consequently, to establish a large ΔΨ under alkaline conditions, K^+^ uptake requirements should be minimal (7–9). However, recent studies demonstrated the requirement for the *S. aureus* Ktr system during low-K^+^ growth under alkaline but not neutral or acidic pH conditions (1), indicating that our understanding of this system is incomplete.

### K^+^ deficiency inhibits *S. aureus* PMF generation.

**FIG 1:**
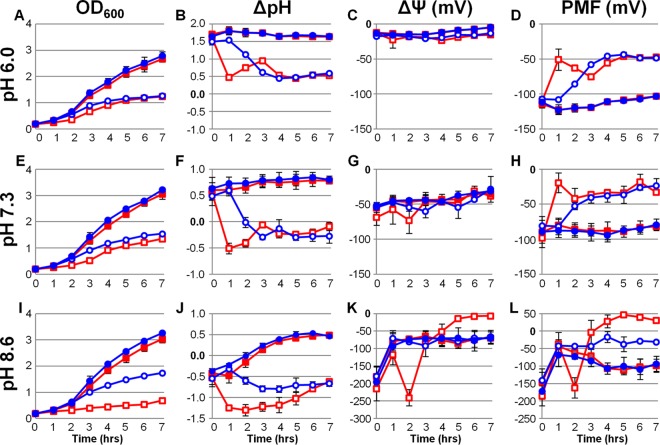
Contribution of K^+^ uptake to the generation of *S. aureus* proton motive force. Wild-type *S. aureus* (blue lines) and its isogenic Δ*ktrC* mutant (red lines) were inoculated to an OD_600_ of 0.2 in K^−^TB with (filled symbols) and without (empty symbols) 10 mM supplemental KCl at pH 6.0 (A to D), 7.3 (E to H), and 8.6 (I to L). Immediately following inoculation and each hour after, OD_600_ (A, E, and I), intracellular and extracellular pH (ΔpH) (B, F, and J), and transmembrane electrical potential (ΔΨ) (C, G, and K) were measured and subsequently calculated. The total proton motive force was calculated from these values (PMF) (D, H, and L). Results generated using pH 6.0 media are averages from four biological replicates (*n* = 4) acquired from two independent experiments with the standard deviations shown. Results generated using pH 7.3 and 8.6 media are averages from at least three independent experiments with the standard deviations shown.

To further assess the role of K^+^ uptake in *S. aureus* metabolism, we measured the individual PMF components during growth with and without the addition of K^+^ at acidic, neutral, and alkaline pH. Cells harboring a plasmid encoding a pH-sensitive green fluorescent protein (GFP) (pHluorin) (10, 11) were cultured in a K^+^-deficient tryptone broth (K^−^TB) at pH 6.0, 7.3, and 8.6. Immediately after and at each hour following inoculation, cell density (optical density at 600 nm [OD_600_]), culture pH, cytoplasmic pH, and ΔΨ measurements were examined. As shown in Fig. 1A, E, and I, growth of the wild-type *S. aureus* strain was inhibited under all 3 pH conditions lacking added K^+^ compared to when the medium was supplemented with 10 mM K^+^. Confirming previous findings in a chemically defined medium (1), growth of the isogenic Δ*ktrC* mutant strain was severely inhibited in alkaline media lacking supplemental K^+^ while only slightly delayed in comparison with the wild type at pH 7.3 and unchanged in acidic K^+^-deficient medium. These data demonstrate the importance of K^+^ uptake for *S. aureus* fitness and illustrate a critical role for the Ktr system under alkaline conditions.

Interestingly, the ΔpH of both the wild type and the Δ*ktrC* mutant dropped over 1 pH unit within 3 h of inoculation in each K^+^-deficient medium (Fig. 1B, F, and J). This was due to rapid acidification of the cytoplasm, as the culture pH remained unchanged from the pH of those supplemented with K^+^ (see Fig. S1 in the supplemental material). The rate of ΔpH drop was markedly higher in the Δ*ktrC* mutant strain, representing the contribution of the Ktr system to maintenance of cytoplasmic pH under these conditions. Importantly, in neutral and alkaline K^+^-deficient media, the extent of cytoplasmic acidification resulted in a negative ΔpH. Under these conditions, ATP synthase activity relies on a large ΔΨ for H^+^ return. To our surprise, the ΔΨ appeared to be largely independent of K^+^ availability, and yet pH-dependent changes of ~25 mV between experiments under each condition were observed (Fig. 1C, G, and K). Additionally, ΔΨ was unchanged between strains; however, a sharp ΔΨ hyperpolarization followed by a rapid depolarization was observed in the Δ*ktrC* mutant at pH 8.6 shortly after inoculation. We speculate that these changes correspond with the inability to release and convert H^+^ on the outer membrane leaflet into a ΔpH via Ktr-mediated K^+^ uptake. As shown in the PMF calculations (Fig. 1D, H, and L), *S. aureus* relies heavily on adequate K^+^ uptake, regardless of environmental pH, to establish the cellular bioenergetics required for growth. Taken together, the data presented in Fig. 1 indicate that limited K^+^ uptake results in the failure to effectively neutralize an acidified cytoplasm caused by the reduced capacity for H^+^ efflux.

10.1128/mSphere.00125-16.1 Figure S1  Culture and cytoplasmic pH measurements. Wild-type *S. aureus* (blue and black lines) and its isogenic Δ*ktrC* mutant (red and gray lines) were inoculated to an OD_600_ of 0.2 in K^−^TB with (filled symbols) and without (empty symbols) 10 mM supplemental KCl at pH 6.0 (A), 7.3 (B), and 8.6 (C). Immediately following inoculation and each hour after, culture pH (dashed lines) and intracellular pH (solid lines) were measured. Results generated using pH 6.0 medium are averages from four biological replicates (*n* = 4) acquired from two independent experiments with the standard deviations shown. Results generated using pH 7.3 and 8.6 media are averages from at least three independent experiments with the standard deviations shown. Download Figure S1, TIF file, 0.3 MB. Copyright © 2016 Gries et al. 2016 Gries et al. This content is distributed under the terms of the Creative Commons Attribution 4.0 International license.

### Metabolic pathways are influenced by K^+^ availability.

**FIG 2:**
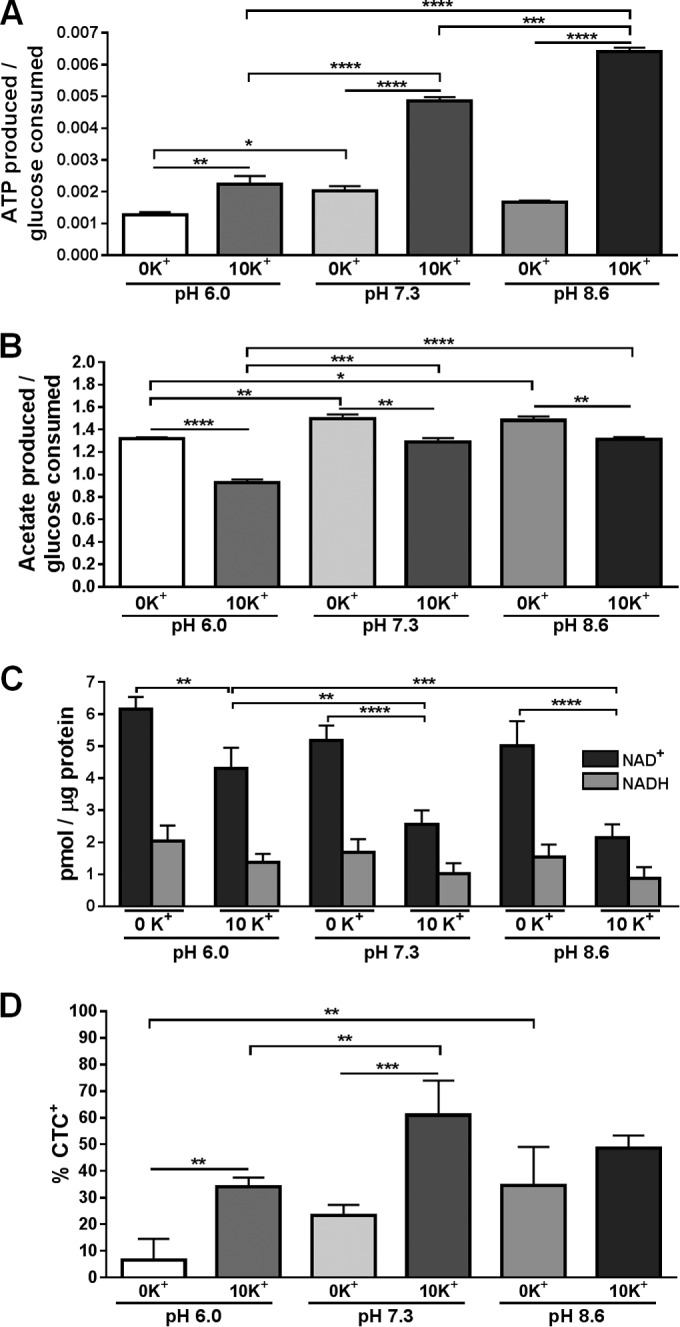
Influence of K^+^ uptake on *S. aureus* metabolism. Wild-type *S. aureus* was inoculated to an OD_600_ of 0.2 in K^−^TB with and without 10 mM supplemental KCl at pH 6.0 (left columns), 7.3 (middle columns), and 8.6 (right columns). Following 6 h of growth, ATP generated per glucose consumed (A), acetate produced per glucose consumed (B), NAD^+^ and NADH levels (C), and the percentage of CTC^+^-stained cells (D) were determined. Results generated from the ATP, glucose, and CTC^+^ analyses are averages from four biological replicates (*n* = 4) acquired from two independent experiments with the standard deviations shown. NAD^+^ and NADH data are averages from three independent experiments with the standard deviations shown. Statistical analysis was performed using a one-way analysis of variance method with Tukey’s multiple-comparison test for *post hoc* analysis (*, *P* < 0.05; **, *P* < 0.01; ***, *P* < 0.001; ****, *P* < 0.0001).

During aerobic growth in the presence of excess glucose, *S. aureus* generates ATP through substrate-level phosphorylation via glycolysis and the phosphotransacetylase-acetate kinase (Pta-AckA) pathway and by oxidative phosphorylation using NADH formed by glycolytic machinery and pyruvate dehydrogenase complex (PDHC) as electron donors (12). The flux of acetyl coenzyme A (CoA) into the tricarboxylic acid (TCA) cycle under these conditions is limited due to carbon catabolite repression (13). To assess the impact of K^+^ availability on the metabolic and energy states of *S. aureus*, wild-type cells and culture supernatants were collected following 6 h of growth under medium conditions described above. We first examined the efficiency at which cells catabolized glucose to produce energy in the form of ATP. As shown in Fig. 2A, intracellular ATP pools varied slightly among experiments under conditions lacking K^+^, while the availability of 10 mM K^+^ significantly increased ATP generation from glucose in a pH-dependent manner. Importantly, cells collected at 6 h are subject to carbon catabolite repression due to sufficient glucose (≥3 mM) remaining under all medium conditions tested (data not shown). This results in funneling of acetyl-CoA toward acetate generation and excretion into the culture medium. To assess the amount of glucose catabolized through the Pta-AckA pathway, we quantified the amount of acetate in the culture supernatants (Fig. 2B). Significantly more acetate was produced per glucose consumed under conditions lacking K^+^. Additionally, acetate generation was pH dependent and significantly greater at neutral and alkaline pH. These data suggest that under aerobic conditions with limited K^+^ availability, carbon flux in *S. aureus* is directed further away from the TCA cycle, toward the Pta-AckA pathway. This is supported by the increased amount of acetate generated despite similar rates of glucose consumption under these conditions (data not shown).

We next examined the status of the oxidative phosphorylation pathway by first measuring cellular NADH and NAD^+^ pools. NADH is oxidized to NAD^+^ at complex 1 of the electron transport chain (ETC) by the enzyme NADH dehydrogenase, and NADH is regenerated by oxidation-reduction reactions within glycolysis, PDHC, and the TCA cycle. As shown in Fig. 2C, cells cultured without K^+^ had significantly greater intracellular NAD^+^ pools and moderately increased NADH levels (*P* = 0.105, 0.093, and 0.089 at pH 6.0, 7.3, and 8.6, respectively) compared to cells cultured in 10 mM K^+^. We speculate that K^+^ deficiency impairs ETC activity, reflected by reduced ATP levels, and stimulates NAD^+^ salvage and/or *de novo* biosynthesis with subsequent reduction to NADH by glycolysis and PDHC to compensate for cellular requirements in reducing equivalents. To test this, we examined ETC activity under these conditions, by staining cells with the fluorescent redox dye 5-cyano-2,3-ditolyl tetrazolium chloride (CTC). CTC can accept electrons from the ETC and is reduced to an insoluble, red fluorescent formazan product, thereby providing a semiquantitative measure of ETC activity. As anticipated, significantly fewer cells were CTC^+^ when cultured for 6 h without K^+^ at pH 6.0 and 7.3 than when cultured with 10 mM K^+^ (Fig. 2D), indicating that K^+^ deficiency affects ETC activity and supporting a role for K^+^ in directing carbon flux toward oxidative phosphorylation.

### Conclusions.

*S. aureus* has the ability to overcome various environmental stresses, and the data shown here reveal a critical role for K^+^ uptake in directing *S. aureus* metabolism under a range of conditions. We have demonstrated that K^+^ uptake deficiency leads to decreased oxidative phosphorylation activity and carbon flux shifted toward substrate-level phosphorylation pathways. This was evident with inefficient ATP production, increased acetate generation per glucose consumed, accumulation of NAD^+^ and NADH species, and decreased CTC^+^ cells in the absence of K^+^. Additionally, we provide evidence that K^+^ uptake in *S. aureus* is required for cytoplasmic pH homeostasis. Rapid acidification of the cytoplasm was observed in cells lacking sufficient K^+^ uptake, resulting in a failure to generate a PMF sufficient for normal growth.

Based on the data presented here, we hypothesize that *S. aureus* Ktr-mediated K^+^ uptake is coupled with ETC H^+^ efflux; insufficient K^+^ transport causes ETC dysfunction and cytoplasmic H^+^ accumulation. Decreased ETC activity results in NAD^+^ and NADH species accumulation and glucose flux shifted toward the AckA-Pta pathway. This is supported by the pH-dependent phenotypes where, under alkaline conditions, establishment of a positive ΔpH requires additional H^+^ extrusion and, consequently, increased K^+^ uptake. Together, these findings further our understanding of the role for K^+^ uptake in *S. aureus*. However, it remains unclear how Ktr-mediated K^+^ uptake in *S. aureus* is energized and the extent to which K^+^ uptake and H^+^ efflux are coupled.

While it has previously been shown that K^+^ uptake plays a role in antibiotic tolerance and *S. aureus* pathogenesis (1), advances in the understanding of ion flux in *S. aureus* will undoubtedly reveal mechanisms by which this versatile pathogen can thrive both within and outside the host. The regulation of K^+^ uptake mechanisms in *S. aureus* is only now becoming realized, and modification of the KtrC protein activity by the recently discovered second messenger c-di-AMP leads to intriguing questions regarding the role of these processes in *S. aureus* physiology (2). Finally, further investigations of the cellular processes affected by K^+^ transport will aid in our understanding of the complex role that this essential macroelement plays in maintaining physiological homeostasis.

### Bacterial strains, plasmid construction, and growth conditions.

*Escherichia coli* DH5α was used for cloning and grown at 37°C in lysogeny broth (LB) with ampicillin (100 µg ⋅ ml^−1^) for selection. DNA ligase and restriction enzymes were obtained from New England Biolabs (Beverly, MA). Plasmids were purified using the Wizard Plus SV Miniprep DNA purification system (Promega Corporation, Madison, WI) and analyzed using Vector NTI (Invitrogen, Carlsbad, CA). The *S. aureus* strain used is a USA300 isolate from a skin and soft tissue infection (see Table S2 in the supplemental material). Codon optimization of pHluorin for *S. aureus* was performed (GeneArt; Invitrogen) based on the GenBank sequence (AF058694.2). The *S. aureus sarA* P1 promoter and *sodA* ribosome binding site were positioned 5′ of pHluorin for constitutive expression in *S. aureus*. The sequence was digested from pHopt with EcoRI and XbaI and then ligated to a similarly digested pCM28, creating plasmid pCG44.

A K^+^-deficient tryptone broth (K^−^TB) was composed, including 10 g ⋅ liter^−1^ Na_2_HPO_4_, 2.5 g ⋅ liter^−1^ glucose, and 2.5 g ⋅ liter^−1^ tryptone, and used for pregrowth and experimental cultures (see Table S1 in the supplemental material). The contaminating K^+^ concentration in this medium was 0.10 mM as determined by flame photometry (Instrumentation Laboratories, Boston, MA). For pregrowth, isolated colonies of *S. aureus* carrying pCG44 or its parental plasmid pCM28 were inoculated in 25 ml K^−^TB (pH 7.3) supplemented to 10 mM KCl with chloramphenicol (10 µg ⋅ ml^−1^) and grown at 37°C and 250 rpm for 16 to 18 h. Cells were washed twice with sterile diH_2_O prior to experimentation.

10.1128/mSphere.00125-16.3 Table S1  K^−^TB formulation Download Table S1, DOCX file, 0.1 MB. Copyright © 2016 Gries et al. 2016 Gries et al. This content is distributed under the terms of the Creative Commons Attribution 4.0 International license.

10.1128/mSphere.00125-16.4 Table S2  Bacterial strains and plasmids Download Table S2, DOCX file, 0.1 MB. Copyright © 2016 Gries et al. 2016 Gries et al. This content is distributed under the terms of the Creative Commons Attribution 4.0 International license.

### Calculating PMF components.

A pHluorin standard curve was generated similarly to methods previously described (10, 11). Briefly, cells were resuspended to an OD_600_ of 1.0 in 200 µl of 0.1 M citric acid, 0.2 M K_2_HPO_4_ buffer from pH 6.0 to 8.5 in a black-bottom 96-well plate (Corning, Inc., NY). One sample set was incubated for 10 min with 2 µM valinomycin and 2 µM nigericin to equilibrate intracellular and extracellular pH. Fluorescence emission at 510 nm with excitation at 410 and 470 nm was then measured using a Tecan 200 Infinite Pro reader (Tecan Group Ltd., Männedorf, Switzerland), and the emission ratios (410/470 nm) were plotted (see Fig. S2A in the supplemental material). A polynomial equation calculated based on the valinomycin/nigericin-treated sample was used to calculate intracellular pH during experimentation.

10.1128/mSphere.00125-16.2 Figure S2  Intracellular pH and membrane potential standard curves. (A) Ratiometric calibration curve of pHluorin. *S. aureus* cells harboring pCG44 were inoculated to an OD_600_ of 1.0 in 200 µl calibration buffer ranging from pH 6.0 to 8.5 without (blue line) and with 2 µM nigericin and 2 µM valinomycin (V/N; red line). Fluorescence emission was measured at 510 nm with excitation at 410 and 470 nm, and the 410/470 ratio was calculated for each pH buffer. A standard curve and polynomial equation were generated from these data. Data are a representative standard curve. (B) Membrane potential calibration curve. *S. aureus* cells harboring plasmid pCG44 were diluted to an OD_600_ of 2.0 in membrane potential calibration buffer with 5 µM valinomycin, 50 µM DiOC_2_(3), and a 0.1 to 100 mM range of KCl in a black-bottom 96-well plate. Fluorescence emission was measured at 505 and 585 nm following excitation at 450 nm. Values were normalized to unstained cells to account for pCG44 fluorescence. An additional DiOC_2_(3)-stained sample containing 5 µM CCCP (solid circles) was added as a depolarized control. Data are a representative standard curve. Download Figure S2, TIF file, 0.1 MB. Copyright © 2016 Gries et al. 2016 Gries et al. This content is distributed under the terms of the Creative Commons Attribution 4.0 International license.

A ΔΨ calibration curve using the carbocyanine dye 3,3′-diethyloxacarbocyanine iodide [DiOC_2_(3)] (*Bac*Light; Molecular Probes) was established similarly to methods previously described (14). Briefly, cells harboring plasmid pCG44 were resuspended to an OD_600_ of 2.0 in 200 µl of ΔΨ calibration buffer containing 0.1 to 100 mM KCl, 5 µM valinomycin, and 50 µM DiOC_2_(3) in a black-bottom 96-well plate. An additional sample containing 5 µM protonophore carbonyl cyanide *m*-chlorophenylhydrazone (CCCP) was used as a depolarized control. Background fluorescence was normalized using unstained samples. Cells were incubated in the dark for 10 min. Fluorescence emission at 505 and 585 nm with excitation at 450 nm was then measured. The 585/505 ratio was determined, subsequent calculation of respective ΔΨ (millivolts) at each KCl value was performed using the Nernst equation, and a linear trendline was derived (see Fig. S2B in the supplemental material).

For experimentation, washed cells from overnight cultures were diluted to an OD_600_ of 0.2 in 50 ml prewarmed K^−^TB in a 500-ml Erlenmeyer flask. Immediately following inoculation, 200 µl of culture was added to a black-bottom 96-well plate and intracellular pH was measured as described above. Culture pH was measured for ΔpH calculation. For ΔΨ measurement, 30 µM DiOC_2_(3) was added to 200 µl of culture concentrated to an OD_600_ of 2.0 in the sample supernatant and incubated for 10 min in a black-bottom 96-well plate. Fluorescence measurements using excitation at 488 nm and emission at 505 and 585 nm were acquired as described above, including normalization for background fluorescence using an unstained control sample.

### Measurement of intracellular ATP, NAD^+^, NADH, and extracellular glucose and acetic acid concentrations.

Quantification of metabolites was performed as previously described (12). Intracellular ATP concentrations were determined using BacTiter-Glo (Promega) according to the manufacturer’s instructions. Intracellular NAD^+^ and NADH concentrations were determined using Fluoro NAD/NADH (Cell Technology) as previously described (12) and normalized to the respective total cellular protein concentration. Extracellular glucose and acetate concentrations were determined using kits purchased from R-Biopharm (Marshal, MI) according to the manufacturer’s instructions as previously described (12).

### CTC staining and flow cytometry.

Cells from 6-h cultures were stained with 5 mM CTC for 30 min prior to flow cytometry analysis. A BD LSR II flow cytometer (Becton, Dickinson, San Jose, CA) was used at a flow rate of 1,000 cells/s. A total of 10,000 events were collected for each sample. Bacteria were discriminated from the background using a combination of forward-scattered light and side-scattered light. CTC emission was detected at 695 ± 40 nm (with a 685-nm long-pass mirror). Raw data were analyzed using FlowJo software.
