# Supplementary figures and images for: Potassium Uptake Modulates Staphylococcus aureus Metabolism
Source: mSphere. 2016 Jun 15;1(3):e00125-16. doi: 10.1128/mSphere.00125-16 (PMC4911797; doi:10.1128/mSphere.00125-16)

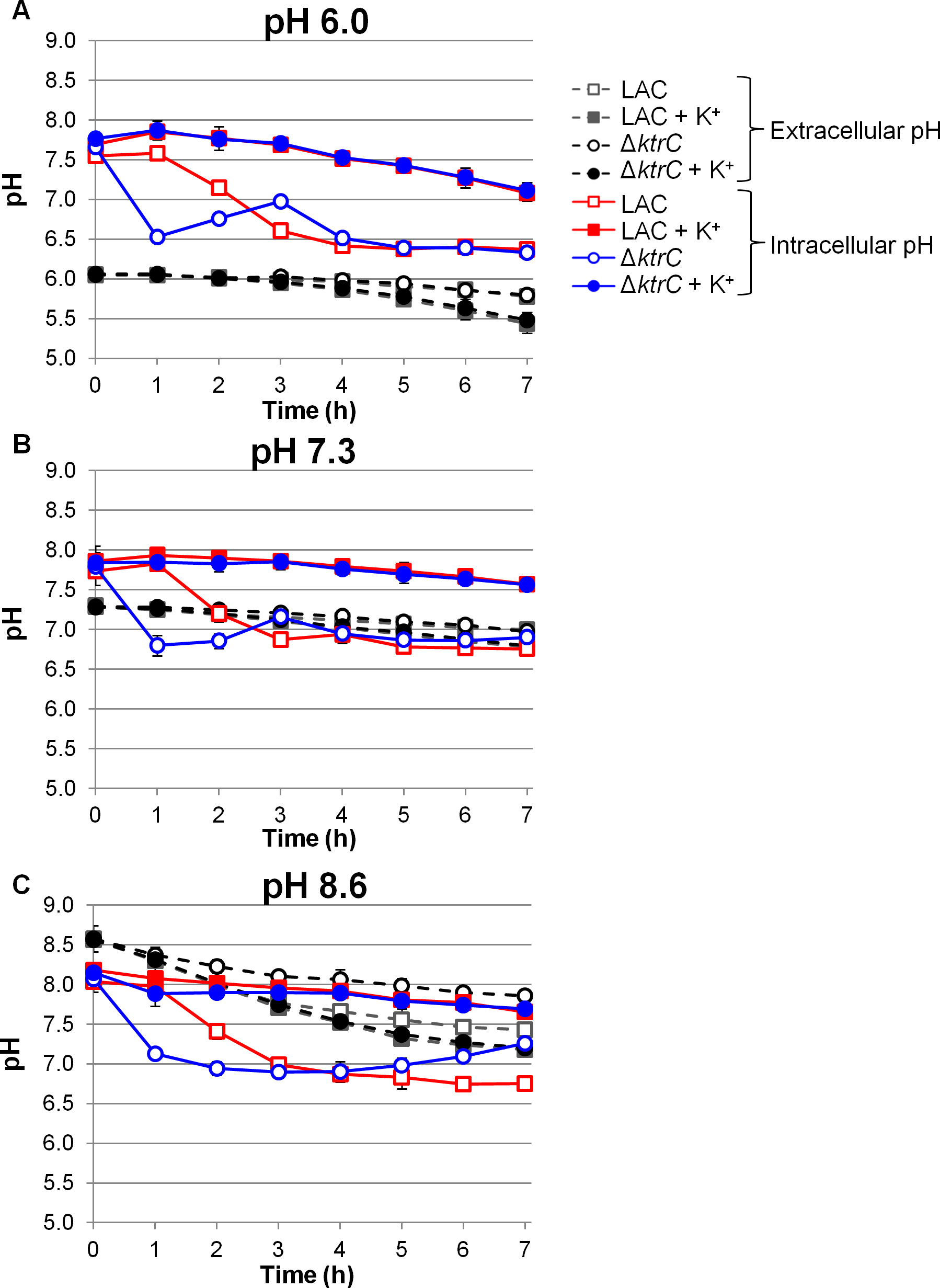

Supplement: Figure S1 [file sph003162105sf1.tif]

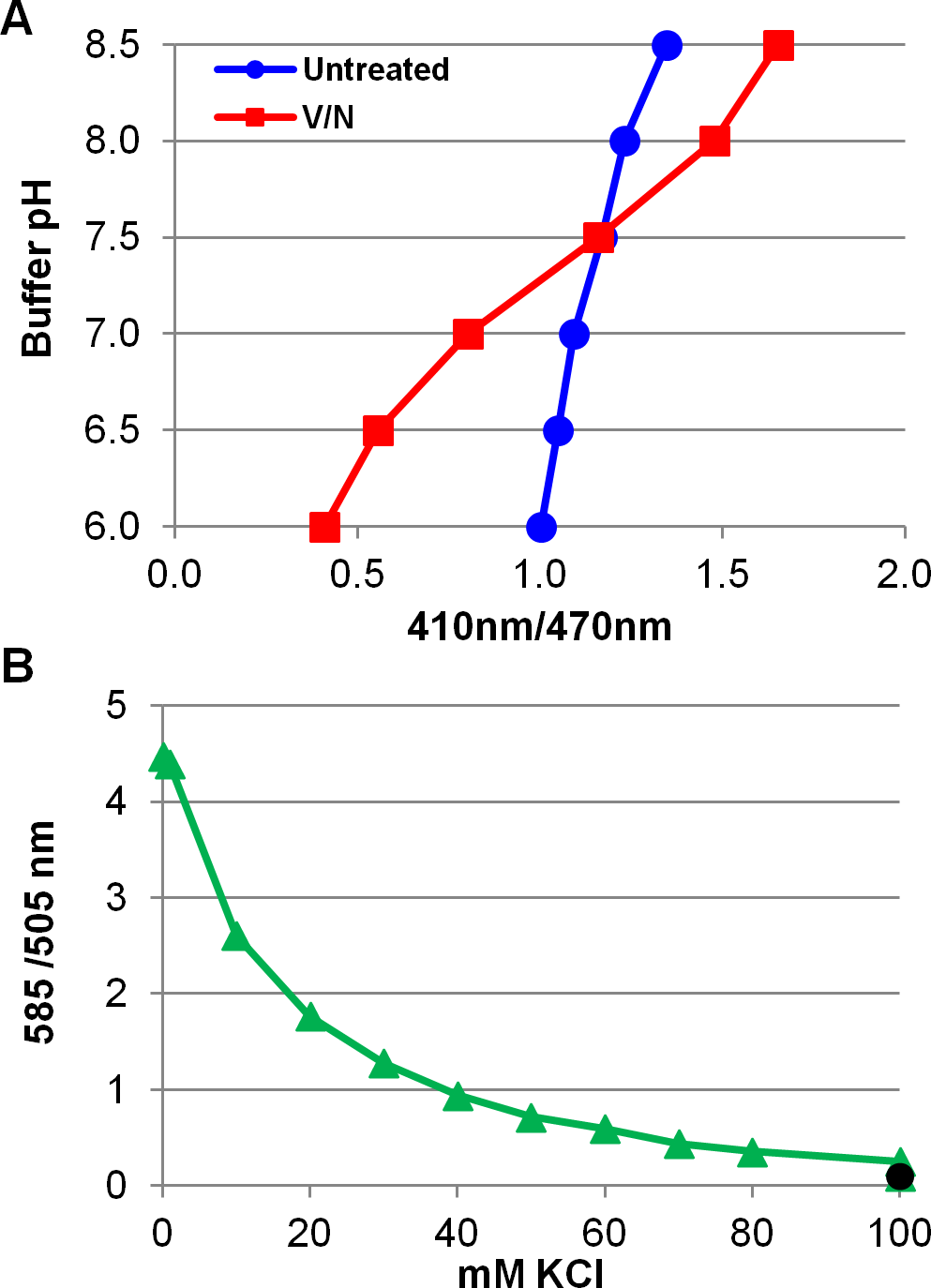

Supplement: Figure S2 [file sph003162105sf2.tif]
