# Supplementary material for: Potassium Uptake Modulates Staphylococcus aureus Metabolism
Source: mSphere. 2016 Jun 15;1(3):e00125-16. doi: 10.1128/mSphere.00125-16 (PMC4911797; doi:10.1128/mSphere.00125-16)
Supplement: Table S1 [file sph003162105st3.docx]

**Supporting Table 1: K^-^TB formulation**

| Group 1 (1x – made fresh) | Group 2 (100x) | Group 3 (100x) |
| --- | --- | --- |
| 10.0 g/L Na_2_HPO_4_ | 1.0 g/L CaCl_2_·2H_2_0 | 0.2 g/L nicotinic acid |
| 2.5 g/L glucose | 0.5 g/L MnSO_4_ | 0.2 g/L thiamine |
| 2.5 g/L tryptone | 0.6 g/L (NH_4_)_2_SO_4_·FeSO_4_·7H_2_O |  |
| 0.5 g/L MgSO_4_·7H_2_0 | 0.6 g/L citric acid |  |

*Adjust pH with concentrated NaOH or HCl and sterile filter (0.2 µM)*
