# Supplementary material for: Potassium Uptake Modulates Staphylococcus aureus Metabolism
Source: mSphere. 2016 Jun 15;1(3):e00125-16. doi: 10.1128/mSphere.00125-16 (PMC4911797; doi:10.1128/mSphere.00125-16)
Supplement: Table S2 [file sph003162105st4.docx]

**Supporting Table 2: Bacterial Strains and plasmids**

| **Strain** | **Characteristics** | **Source** |
| --- | --- | --- |
| LAC | Wild-type *S. aureus* CA-MRSA (USA300) isolate | ([4](#_ENREF_4)) |
| KB7005 | LAC Δ*ktrC* | ([1](#_ENREF_1)) |
| RN4220 | Highly-transformable restriction-deficient strain | ([2](#_ENREF_2)) |
|  |  |  |
| **Plasmids**^a^ | **Characteristics** | **Source** |
| pHopt | P*sarA*::pHluorin (codon-optimized for *S. aureus*) | Invitrogen |
| pCM28 | Expression plasmid, *ampR*, *chmR* | ([3](#_ENREF_3)) |
| pCG44 | pCM28 with P*sarA*::pHluorin | This study |

^a^ Antibiotic resistance abbreviations: *ampR*, ampicillin (bla), *chmR*, chloramphenicol

1. Gries, C. M., J. L. Bose, A. S. Nuxoll, P. D. Fey, and K. W. Bayles. 2013. The Ktr potassium transport system in Staphylococcus aureus and its role in cell physiology, antimicrobial resistance and pathogenesis. Molecular microbiology 89:760-773.

2. Kreiswirth, B. N., S. Lofdahl, M. J. Betley, M. O'Reilly, P. M. Schlievert, M. S. Bergdoll, and R. P. Novick. 1983. The toxic shock syndrome exotoxin structural gene is not detectably transmitted by a prophage. Nature 305:709-712.

3. Pang, Y. Y., J. Schwartz, M. Thoendel, L. W. Ackermann, A. R. Horswill, and W. M. Nauseef. 2010. agr-Dependent interactions of Staphylococcus aureus USA300 with human polymorphonuclear neutrophils. Journal of innate immunity 2:546-559.

4. Voyich, J. M., K. R. Braughton, D. E. Sturdevant, A. R. Whitney, B. Said-Salim, S. F. Porcella, R. D. Long, D. W. Dorward, D. J. Gardner, B. N. Kreiswirth, J. M. Musser, and F. R. DeLeo. 2005. Insights into mechanisms used by Staphylococcus aureus to avoid destruction by human neutrophils. Journal of immunology 175:3907-3919.
